# Relationship Between Circadian Strain, Light Exposure, and Body Mass Index in Rural and Urban Quilombola Communities

**DOI:** 10.3389/fphys.2021.773969

**Published:** 2022-01-26

**Authors:** Débora Barroggi Constantino, Nicoli Bertuol Xavier, Rosa Levandovski, Till Roenneberg, Maria Paz Hidalgo, Luísa K. Pilz

**Affiliations:** ^1^Laboratório de Cronobiologia e Sono, Hospital de Clínicas de Porto Alegre (HCPA)/Universidade Federal do Rio Grande do Sul (UFRGS), Porto Alegre, Brazil; ^2^Psychiatry and Behavioral Sciences Program (PPG) em Psiquiatria e Ciências do Comportamento, Universidade Federal do Rio Grande do Sul (UFRGS), Porto Alegre, Brazil; ^3^Psychiatry and Behavioral Sciences Program (PPG) Avaliação e Produção de Tecnologias para o Sistema Único de Saúde (SUS), Grupo Hospitalar Conceição (GHC), Porto Alegre, Brazil; ^4^Psychiatry and Behavioral Sciences Program (PPG) Saúde Coletiva, Universidade Federal do Rio Grande do Sul (UFRGS), Porto Alegre, Brazil; ^5^Institute of Medical Psychology – Ludwig Maximilian University (LMU), Munich, Germany

**Keywords:** actimetry, obesity, intradaily variability, chronobiology, rest-activity rhythms, levels of urbanization, relative amplitude

## Abstract

Industrialization has greatly changed human lifestyle; work and leisure activities have been moved indoors, and artificial light has been used to illuminate the night. As cyclic environmental cues such as light and feeding become weak and/or irregular, endogenous circadian systems are increasingly being disrupted. These disruptions are associated with metabolic dysfunction, possibly contributing to increased rates of overweight and obesity worldwide. Here, we aimed to investigate how activity-rest rhythms, patterns of light exposure, and levels of urbanization may be associated with body mass index (BMI) in a sample of rural and urban Quilombola communities in southern Brazil. These are characterized as remaining social groups who resisted the slavery regime that prevailed in Brazil. Quilombola communities were classified into five groups according to their stage of urbanization: from rural areas with no access to electricity to highly urbanized communities. We collected anthropometric data to calculate BMI, which was categorized as follows: from ≥ 18.5 kg/m^2^ to < 25 kg/m^2^ = normal weight; from ≥ 25 kg/m^2^ to < 30 kg/m^2^ = overweight; and ≥ 30 kg/m^2^ = obese. Subjects were asked about their sleep routines and light exposure on workdays and work-free days using the Munich Chronotype Questionnaire (*N* = 244 included). In addition, we analyzed actimetry data from 121 participants with seven consecutive days of recordings. Living in more urbanized areas and higher intradaily variability (IV) of activity-rest rhythms were associated with an increased risk of belonging to the overweight or obese group, when controlling for age and sex. These findings are consistent with preclinical data and point to potential strategies in obesity prevention and promotion of healthy metabolic profiles.

## Introduction

The prevalence of obesity has tripled since 1975 and constitutes a major clinical and research challenge worldwide (Abarca-Gómez et al., 2017). In 2016, more than 1.2 billion adults were overweight and nearly 650 million adults were obese (World Health Organization [WHO], 2020). Increased body mass index (BMI) is associated with many comorbidities, such as cardiovascular diseases, diabetes, osteoarthritis, and some forms of cancers (Haslam and James, 2005; Wolin et al., 2010). Weight gain results from multiple conditions and complex interactions between the factors that still need better characterization (Blüher, 2019). Disruption of circadian rhythms, which may be driven by societal constraints (e.g., work schedules), has been described as one factor potentially contributing to weight gain (Roenneberg et al., 2012).

To synchronize (entrain) to the rhythmic environment, the central circadian clock in the suprachiasmatic nucleus (SCN) predominantly uses light as a *zeitgeber* (environmental signal specific for entrainment; Roenneberg and Merrow, 2007; LeGates et al., 2014). The SCN provides an internal rhythmic milieu that is used by the cellular clocks in tissues and organs to entrain to the light-dark cycle of the environment (*via* the SCN), and also to other clocks of the peripheral circadian system. This system modulates all aspects of physiology (e.g., body temperature, hormone secretion, feeding behavior, alertness, and cognitive function; Mohawk et al., 2012; Astiz et al., 2019), thereby also coordinating metabolism on a daily basis (Marcheva et al., 2013; Reinke and Asher, 2019). Notably, peripheral clocks, for example, in the liver, can also be entrained by food intake (Damiola et al., 2000; Panda and Hogenesch, 2004; Buijs et al., 2013).

Human circadian organization is affected by current lifestyles and behaviors in modern societies, including weak zeitgeber signals (due to less time spent outdoors and artificial light after dusk; Roenneberg and Merrow, 2016). Timing, duration, and intensity of light exposure have strong effects on health and behavioral outcomes (Gonzalez, 2018; Rumanova et al., 2020); exposure to weak (low amplitude) light-dark cycles, for example, is associated with metabolic changes and weight gain (Borniger et al., 2014). Weak or irregular light exposure is often associated with circadian misalignment (e.g., in shift workers or simply early work start times that lead to use alarm clocks), which itself is linked to obesity (Roenneberg et al., 2012; Liu et al., 2018). Disturbances of daily rhythmicity have been related to cardiovascular disease, diabetes, aging, and obesity (Sohail et al., 2015; Sletten et al., 2020). When rodents are under forced desynchrony (modeling circadian disruption), glucose metabolism and insulin sensitivity become impaired (Karatsoreos et al., 2011; de Oliveira et al., 2019). However, the precise mechanisms linking circadian disruption with adverse metabolic consequences are not fully understood.

Few studies have evaluated the relationship between circadian rhythms, light exposure, and body weight in clinical, epidemiological, or field studies. Yet, identifying modifiable behaviors or aspects promoting weight gain is essential to understand the underlying mechanisms and to devise effective interventions. Here, we report the results of studying the association between BMI, light exposure, and activity-rest rhythms in Quilombola communities in southern Brazil. These are characterized as remaining social groups established in the past to escape or resist slavery (and slavery remnants) in Brazil with an established ethnic identity and culture. These communities, already described in a previous study (Pilz et al., 2018), have varied histories of access to electricity and live at different stages of urbanization and, therefore, represent a unique opportunity to study the association between circadian misalignment and metabolic factors under the conditions brought by modern industrialized societies. We hypothesized that later phases of entrainment, irregular light exposure, and higher levels of urbanization are associated with overweight or obesity.

## Materials and Methods

### Study Population

**FIGURE 1:**
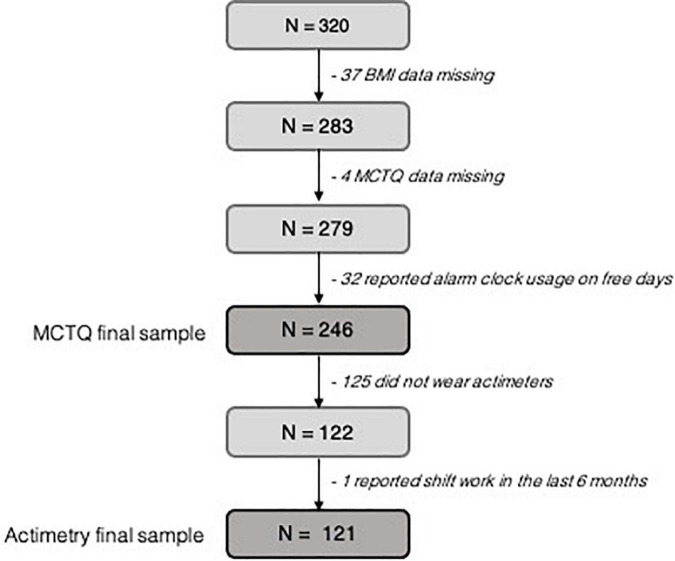
Flowchart of the participants recruitment and selection process. White squares represent final sample size.

Participants were recruited between March 2012 and November 2019 from rural and urban Quilombola communities in southern Brazil. A number of 320 participants (60% women) aging from 16 to 92 years were enrolled in this cross-sectional study. Sociodemographic data, anthropometry, actimetry, and sleep variables were collected. Exclusion criteria included not being able to provide necessary information, having done night shift work in the last 6 months before data collection or having used an alarm clock on work-free days. Since only two subjects with questionnaires’ valid data were underweight, they were also excluded. A number of 244 subjects were included in questionnaire analyses and 121 in actimetry data analyses (see Figure 1).

We used a set of previously validated questionnaires, and interviewers were trained to adapt questions to the level of the participants’ understanding. Additionally, height and weight were measured with a tape measure and a digital scale. BMI was calculated as weight (kg)/height (m)^2^ and categorized as follows: from ≥ 18.5 kg/m^2^ to < 25 kg/m^2^ = normal weight; from ≥ 25 kg/m^2^ to < 30 kg/m^2^ = overweight; and ≥ 30 kg/m^2^ = obese, according to the recommendation of the World Health Organization. On the few occasions in which data could not be directly collected, self-reported values were used instead.

### Ethics

The study was approved by the Ethics Committee of the Hospital de Clínicas de Porto Alegre (project numbers 2019-0218, 2015-0568, and 2011-0502). All participants provided written informed consent, and all study procedures were conducted according to the Declaration of Helsinki. In cases of illiterate participants, an informed consent witnessed by another family member, or the community leader, was collected. When participants were younger than 18 years, parents gave their consent.

### Assessment of Daily Rhythmicity

Participants were asked to wear an actimeter (ActTrust Condor™, Actiwatch-2 Philips Respironics) on their non-dominant wrist for at least 30 consecutive days to characterize their activity-rest rhythms and light exposure. Data were binned in 10-min epochs for analysis. For this study, we included actimetry records of 7 consecutive days (*N* = 121) with a maximum of 4 h of missing data per day. Off-wrist periods (non-wear) were detected using ChronoSapiens software (Roenneberg et al., 2015). These were identified as stretches of at least 10 consecutive zeros, confirmed by visual inspection and set to NA (not available). Activity and light recordings collected using ActTrust were normalized to be comparable to Actiwatch-2. Supplementary Material includes plots of data with no transformation. Considering the smaller sample size, we did not run comparison analyses.

To characterize rhythms of activity and light, we used *cosinor* analysis (Díez-Noguera, 2013). This test provides the following parameters: *mesor* (midline estimating statistic of a rhythm), *amplitude* (difference between the maximum and minimum value of a rhythmic variable), and *acrophase* (time at which a variable reaches its peak, i.e., peak of the fitted curve). We computed *cosinor* parameters using the R package “psych” (Revelle and Condon, 2019).

Daily rhythms were also analyzed by non-parametric circadian rhythm analyses (NPCRA) (Van Someren et al., 1999), which include the following:

(a) interdaily stability (IS) indicates how constant or stable the 24-h rhythmic pattern is across days. It ranges from 0 to 1; higher values indicate more stable rhythms;
(b) intradaily variability (IV) estimates rhythm fragmentation by reflecting transitions between rest and activity and light and dark. The IV would reach near 0 for a perfect sine wave and be about 2 for Gaussian noise; higher values indicate more fragmented rhythms;
(c) M10: mean of measurements over the 10 consecutive hours with the highest values of a daily profile (24-h mean wave);
(d) L5: average of measurements over the 5 consecutive hours with the lowest values of a daily profile (24-h mean wave);
(e) relative amplitude (RA): difference between M10 and L5, divided by their sum.

We also computed and plotted the median and mean daily profiles of light exposure and activity-rest by BMI categories. These were calculated at the individual level, and median group profiles were then computed from the individual daily profiles.

### Sleep Variables

The Brazilian Portuguese version of the Munich Chronotype Questionnaire (available on http://thewep.org/documentations/mctq) was used to assess sleep–wake patterns and natural light exposure on work and work-free days (MCTQ, Roenneberg et al., 2003). MCTQ-derived variables showed good correspondence with the estimates of sleep derived from actimetry in rural Quilombola communities (Pilz et al., 2018). The following variables were computed from the MCTQ data: midpoint of sleep on free days (MSF; as a marker of chronotype), sleep duration on workdays and work-free days, social jetlag (SJL) (measured by subtracting the midpoint of sleep on work-free days from midpoint of sleep on workdays), and the amount of time spent outdoors on workdays and free days.

### Statistical Analyses

Data are reported as means and standard deviations or median and interquartile range, when appropriated. Normality was tested using Shapiro–Wilk and visual inspection of histograms. Since most variables were considered not normally distributed, we used Kruskal–Wallis test followed by Dunn’s test (Sidak correction) to compare parameters derived from the MCTQ or from the actimeter recordings between BMI categories (normal weight, overweight, and obese). Effect sizes and confidence intervals were computed as epsilon-squared (Kelley, 1935) using the R package “*rcompanion”* (Mangiafico, 2021) and as eta-squared (Tomczak and Tomczak, 2014) using the R package “*rstatix”* (Kassambara, 2020). Data were plotted using “*ggplot2”* (Wickham, 2016).

Significant relationships between actimetry- and MCTQ-derived variables and BMI (dependent variable) were further investigated using Poisson regression, and also the association between levels of urbanization and BMI. We used the R package “*jtools”* (Long, 2019) for assessing Poisson results. Since our sample has different sizes for subjective and objective data, we used one model for parameters derived from MCTQ and one model for actimetry data. We also analyzed the association between data derived from the actimeters and BMI in two models: one for activity and the other for light variables. The variables were selected based on both the results from Kruskal–Wallis tests (difference between normal BMI and overweight) and effect sizes (moderate). For the final actimetry model, we selected the variables that were significant in the previous models. Models were controlled for age and sex and used a robust estimator (HC0). We assessed multicollinearity using variance inflation factor (VIF) test and excluded variables when collinearity was severe (VIF > 5; in our models VIF < 2 for all independent variables) (Kutner et al., 2004). Finally, we tested the linearity of parameters included in the models using the Wald test. Since SJL did not meet the linearity assumption, we chose to categorize this variable.

## Results

### Characteristics of the Study Population

General characteristics of the sample are shown in Table 1. In the MCTQ dataset, the median of age was 45 years [Q_1_–Q_3_: 28–58] and 144 (59%) participants were women. In the actimetry dataset, the median of age was 49 years [Q_1_–Q_3_: 29–61] and 77 (64%) were women. Most participants had only completed primary school. A total of 32.1% of participants were overweight (BMI ≥ 25 kg/m^2^ to < 30 kg/m^2^) and 29.6% were obese (BMI > 30 kg/m^2^).

**TABLE 1:** Sample characteristics.

	MCTQ (*N* = 244)		Actimetry (*N* = 121)	
	N	%	n	%
No electricity n (%)	29	11.7	28	23.1
<5 years n (%)	14	5.7	11	9.0
>20 years n (%)	82	33.3	34	28.0
≥30 years n (%)	98	39.8	35	29.9
Urban n (%)	21	8.5	13	10.7
**Schooling: n (%)**				
Illiterate	31	12.7	18	14.8
Primary school incomplete (1st–4th grade)	97	39.8	47	19.1
Primary school incomplete (5th–7th grade)	39	16.0	18	14.9
Primary school complete	17	7.0	6	4.9
High school incomplete	12	4.9	6	4.9
High school complete	12	4.9	6	4.9
Undergraduate incompleteor graduate degree	2	0.01	0	0.0
Not reported	33	13.5	20	16.5
**BMI status**				
Normal weight: n (%)	92	37.3	49	40.4
Overweight: n (%)	79	32.1	34	28.0
Obese: n (%)	73	29.6	38	31.4
Median BMI [Q_1_–Q_3_ ]	27 [23–30]		26 [23–31]	

### MCTQ Variables and Level of Urbanization According to Body Mass Index Groups

Comparisons of MCTQ variables between BMI groups are provided in Supplementary Table 2. Overall, we did not find any significant differences.

To examine the association of MCTQ variables and levels of urbanization with the odds of being overweight or obese, we also used a Poisson regression model adjusted for age and sex. To that end, we divided subjects into two groups: normal (BMI ≥ 18.5 kg/m^2^ to < 25 kg/m^2^) and overweight or obese (BMI > 25 kg/m^2^). In model 1 (see Table 2), living in highly urbanized communities was associated with increased odds of being overweight or obese (*PR*: 1.63 [1.05–2.53]). SJL was not associated with the outcomes in this sample.

**TABLE 2:** Poisson regression (model 1): variables derived from questionnaires associated with overweight or obesity (*N* = 244).

	PR	95% CI	*p*-value
Intercept	0.311	0.199–0.487	0.000
Age	1.004	0.998–1.010	0.194
Sex (F)	1.545	1.231–1.938	**0.000**
No electricity	1.000	0.535–1.871	0.999
<5 years electricity	1.000	0.534–1.870	0.999
>15 years electricity	1.171	0.776–1.769	0.452
≥30 years electricity	1.413	0.927–2.155	0.108
Urban	1.625	1.045–2.527	**0.031**
SJL (>1 h)	0.886	0.669–1.173	0.398

*Bold values represent statistically significant values.*

### Rest-Activity and Light Exposure Patterns According to Body Mass Index Groups

**FIGURE 2:**
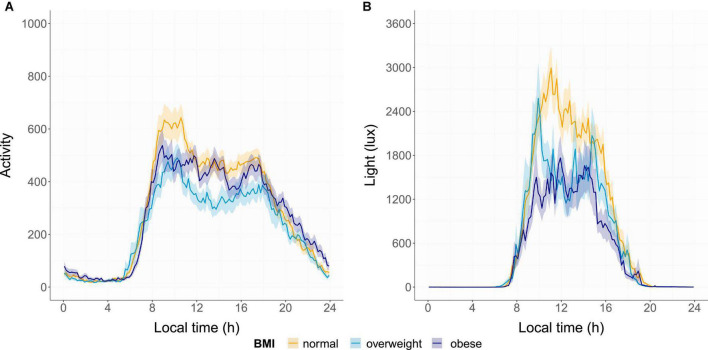
Mean activity **(A)** and light **(B)** profiles of each group. The means (bold line) and standard error (shadow) are presented for the normal-weight group (orange), overweight group (light blue), and obese group (dark blue).

The mean group profiles of light exposure and activity by BMI are shown in Figure 2. Median profiles are shown in Supplementary Figure 1.

**FIGURE 3:**
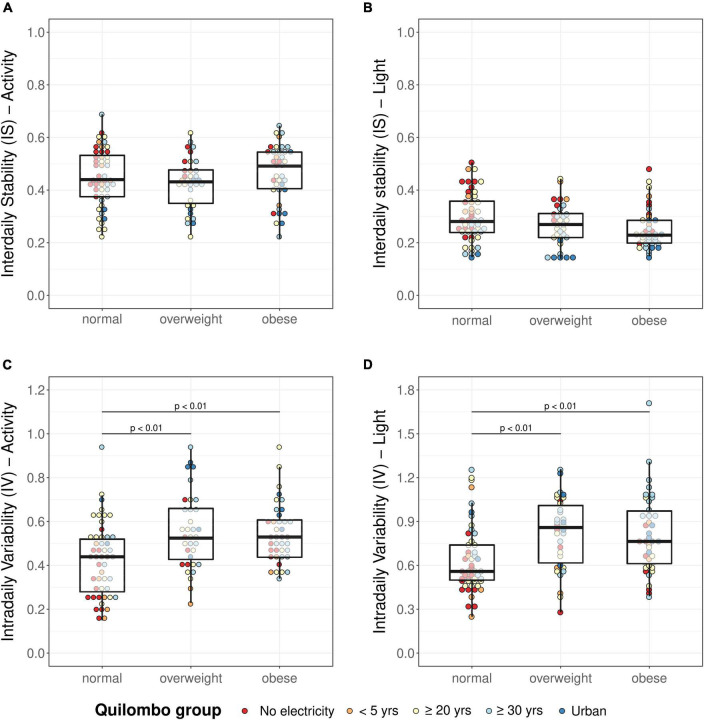
Activity IS **(A)**, activity IV **(C)**, light IS **(B)**, and light IV **(D)** in normal-weight, overweight, and obese groups. Analyses were performed using the Kruskal–Wallis test followed by Dunn’s test. IS, Interdaily stability; IV, intradaily variability; *p*-values according to Dunn’s test for multiple comparisons, adjusted with Sidak method.

As shown in Figure 3, the IV derived from actimetry was different between groups (IV, KW: χ^2^ = 13.49, *p* < 0.01), being higher in both the overweight and the obese group, compared with the normal-weight group. There was no difference between groups in IS derived from actimetry (IS, KW: χ^2^ = 2.67, *p* = 0.26).

Regarding light exposure, the IV was different between groups (KW: χ^2^ = 16.16, *p* < 0.001), being higher in the overweight and obese group as compared to the normal group. Light IS was not significantly different between groups (KW: χ^2^ = 5.48, *p* = 0.06).

**FIGURE 4:**
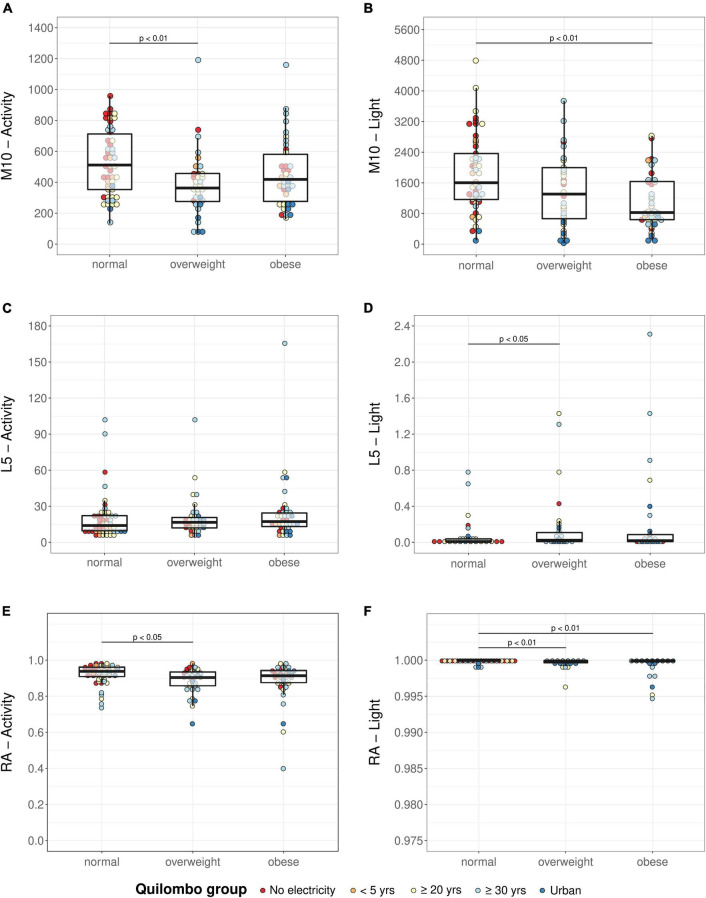
Activity M10 **(A)**, activity L5 **(C)**, activity RA **(E)**, light M10 **(B)**, light L5 **(D)**, and light RA **(F)** in normal-weight, overweight, and obese groups. Analyses were performed using the Kruskal–Wallis test followed by Dunn’s test. M10: mean activity or light exposure of the 10 consecutive hours with the highest values of a daily profile; L5: average activity or light exposure of the 5 consecutive hours with the lowest values of a daily profile; RA, relative amplitude; *p*-values according to Dunn’s test for multiple comparisons, adjusted with Sidak method.

Figure 4 represents M10, L5, and RA of activity and light. Differences were detected in M10 activity between groups (KW: χ^2^ = 9.20, *p* < 0.05): the overweight group had lower values compared with the normal-weight group. Although there was no difference in activity L5 between groups (KW: χ^2^ = 2.47, *p* = 0.29), differences were detected in activity RA (RA; KW: χ^2^ = 8.78, *p* < 0.05): the overweight and obese groups showed lower values.

Regarding light recordings, both M10 (KW: χ^2^ = 12.30, *p* < 0.01) and L5 (KW: χ^2^ = 9.29, *p* < 0.01) were different between groups: light M10 was significantly higher and light L5 was significantly lower in the normal-weight group compared with the obese and overweight, respectively. This indicates higher exposure to light during the day and lower during the night in the normal group compared with the other groups.

Supplementary Figures 2, 3 show NPCRA data split by actimeter brand (Actiwatch-2 vs. ActTrust). We did not run comparison tests considered the smaller sample size. Group mean profiles are shown in Supplementary Figures 4, 5. Since light RA had a narrow range due to the often-low values of light L5, we also plotted light RA as (log10(M10 + 10) − log10(L5 + 10))/(log10(M10 + 10) + log10(L5 + 10)), which is shown in Supplementary Figure 7.

There were also significant differences between groups in the MESOR of activity and light (see more details in Supplementary Material).

Effect sizes and their confidence intervals (by bootstrap) for all Kruskal–Wallis tests, expressed as epsilon-squared (ε^2^) and eta-squared (η^2^), are shown in Supplementary Table 3. They were interpreted as being small to moderate.

To examine the association of variables derived from actimetry with the odds of being overweight or obese, we also used Poisson regressions adjusted for age and sex. Among the variables derived from actimetry tested in model 2 (see Table 3), we found that higher IV of activity is associated with higher odds of belonging to the overweight or obese group (*PR*: 3.04 [1.43–6.47]. As in model 1 (Table 2), the probability of being in the overweight or obese group was significantly higher for women. The separate models with activity and light variables are available in Supplementary Tables 4, 5. The final model additionally adjusted for actimeter brand (ActTrust vs. Actiwatch-2) is also available in Supplementary Table 6.

**TABLE 3:** Poisson regression (model 2): variables derived from actimetry associated to overweight or obesity (*N* = 121).

	PR	95% CI	*p*-value
Intercept	0.125	0.064–0.244	0.000
Age	1.005	0.997–1.012	0.227
Sex (F)	1.687	1.139–2.497	**0.009**
IV (light)	1.632	0.990–2.690	0.055
IV (activity)	3.041	1.429–6.472	**0.004**

*Bold values represent statistically significant values.*

## Discussion

Our study investigated the association of daily behavior and levels of urbanization with BMI. The results show that both activity and light are more fragmented (i.e., higher values of IV) in the overweight or obese group than in the normal-weight participants. Night-time light exposure (light L5) was positively and day-time light exposure (light M10) was negatively associated with having higher than normal BMI. Furthermore, living in more urbanized areas was associated with an increased risk of belonging to the overweight or obese group.

Contrary to previous findings of studies in other populations (Roenneberg et al., 2012; Parsons et al., 2015; [Bibr B41][Bibr B35]), SJL and chronotype were not associated with BMI in our sample: here, we used MSF as a marker of chronotype and we did not detect differences in phase of entrainment or SJL between BMI groups either. The lack of a significant difference in this instance may be related to the low levels of SJL in our rural sample, which may not be sufficient to impose a risk. Additionally, SJL reflects circadian misalignment in the context of work structures normally seen in urban or industrialized societies (workdays vs. free days). Other metrics of circadian strain could better show how challenges to the system may be a risk factor to obesity.

In fact, we did see differences in IV and RA between BMI groups. The IV of activity and light was significantly higher in the overweight and obese groups, compared with the normal-weight group, which indicates that higher fragmentation of daily rhythms associates with overweight and obesity. This result supports those of Garaulet et al. (2017), who found highly fragmented activity rhythms associated with obesity and central adiposity in a sample of adolescents and also with an earlier study that suggested higher fragmentation as a predictor of lower weight loss in overweight and obese women undergoing a weight-reduction program (Bandin et al., 2014). Other large epidemiological studies have shown a positive association between obesity, diabetes, metabolic risk, and fragmentation of activity rhythms in urban populations (Van den Berg et al., 2008; Luik et al., 2013; Sohail et al., 2015). Our study adds to the mounting evidence by showing that activity IV also correlates with BMI in a sample comprised of subjects at different urbanization stages. The higher activity and light variability do not seem to be an artifact produced by different actimeter brands (see Supplementary Figure 2). Even adjusting model 2 to actimeter brand, activity was still significantly associated with being overweight or obese.

Few studies have investigated the associations of stability in activity rhythms (measured by IS) with BMI; some of them found significant associations (Luik et al., 2013; Sohail et al., 2015), whereas others did not (Cespedes Feliciano et al., 2017). In our sample, we did not find any significant differences in the IS between groups.

In addition to the higher fragmentation of light exposure and activity rhythms, in our study, lower RA of activity was related to overweight or obesity. RA is calculated as the difference between M10 and L5, normalized by their sum (Van Someren et al., 1999). Lower values indicate a less robust 24-h activity-rest or light-dark pattern, reflecting both lower activity and light during the wake periods and/or higher activity and light during the rest phase. According to the existing literature, this flattening of the rest-activity rhythm indicates that the circadian pacemaker is less entrained to its zeitgebers, correlating with metabolic disruption (Hsieh et al., 2010; Wright et al., 2013). As expected, the overweight group was less active during the day (i.e., had a lower M10), even though the L5 of activity was the same across groups. Although the M10 of activity is lower in the obese group, it was not significantly different when compared to the normal-weight participants. This may be related to the fact that there is a higher proportion of women in the obese group. In a study conducted by Cooper et al. (2000), it was shown that the differences in activity levels between obese and non-obese participants were greater in men than women. Among NPCRA estimates, RA has been widely investigated as a factor inversely correlated with conditions such as psychiatric disorders, cognitive malfunctioning, and metabolic dysfunction (Ng et al., 2015; Cespedes Feliciano et al., 2017; Lyall et al., 2018). Although we observed the inverse association of RA (of both activity and light) with BMI, such relationship did not persist in the multivariate model, possibly due to the low sample size (data not shown). Furthermore, plots and descriptive statistics suggest similar patterns across groups regardless of the actimeter brand except for M10 and RA of activity. M10 and RA of activity seem to be lower in obese compared with the normal group only among those wearing ActTrust and similar in those wearing the Actiwatch-2. ActTrust is more sensitive to movement, but these differences may also reflect the characteristics of both samples collected with each actimeter, since the sample collected using ActTrust was larger and more varied in terms of urbanization (and therefore in variability measures): no electricity and urban groups data were collected using ActTrust. Even if causal relationships underlying these associations are still not clear, there is growing evidence that simple environmental interventions, such as keeping regular light exposure, feeding, and physical activity times, may improve metabolic health outcomes (Dowling et al., 2005; Van Someren and Riemersma-Van Der Lek, 2007).

The NPCRA estimates for light exposure indicate that a weaker signal and irregular patterns may be related to adverse metabolic consequences and are consistent with recent findings in the literature (McFadden et al., 2014; Wyse et al., 2014; Gerhart-Hines and Lazar, 2015). In mammals, light is the main cue for entraining internal circadian rhythms to environmental cycles. The lack of a strong zeitgeber contrast between day and night leads to advancing extreme early types and delaying all other chronotypes (Papatsimpa et al., 2021). The resulting interaction with local time leads to sleeping and eating at the wrong circadian times, constituting potential mediators linking light exposure to BMI, as shown in both humans and rodents (Fonken et al., 2013; Shi et al., 2013; Plano et al., 2017). Several other studies report that daytime bright light therapy improves glycemic control in patients with diabetes (Versteeg et al., 2017), reduces insulin resistance, body weight, and fat mass (Sene-Fiorese et al., 2015), and improves carbohydrate metabolism (Dunai et al., 2007; Danilenko et al., 2013). Our results support the potential of strategies aimed at adjusting light exposure and promoting circadian organization for preventing obesity.

Another important aspect to be discussed regarding the development of obesity is the changes in lifestyle brought by industrialization that may impact health and increase risk for metabolic diseases. The urban environment comprises factors that affect circadian rhythms and health, such as working in night shifts, prolonged exposure to artificial light, stress, pollution, SJL, and poor diets (Wyse et al., 2014; Pot, 2018; Benedito-Silva et al., 2020). One study that compares Brazilian communities with different levels of urbanization demonstrated that urban dwellers had higher BMI and higher levels of insulin, fasting glucose, and insulin resistance (Martins et al., 2020). Here, we found an association between higher levels of urbanization and increased risk of being overweight or obese. One of the factors explaining these results may be the occupations in rural settings. Since their economy is mostly based on agricultural jobs, they have higher levels of activity during the day. Future studies are needed to investigate how levels of physical activity during work could be a factor contributing to the differences in BMI. Another contributing factor could be the difference in light exposure between some of these communities (Pilz et al., 2018). Reduced natural daylight exposure and increased levels of nocturnal light are common features of urbanized societies and may disrupt circadian rhythmicity, contributing to the association between global urbanization and obesity (Rybnikova et al., 2016). Since we observed a significant association of light at night or low light exposure during the day with overweight or obesity, this may be considered as one of the driving forces behind metabolic diseases. Finally, eating habits were greatly changed by urban settings in many aspects, such as timing of eating, frequency of meals, and diet quality. All these factors may be associated with body weight as well (Pot, 2018).

The main strengths of the current work include the use of objective measures to assess daily behavior. Furthermore, we considered some important covariates and potential confounders such as age, sex, and use of alarm clocks, which may influence the calculation of MSF and SJL. Finally, we could compare many communities that differ in access to electricity and urbanization, which represents a unique opportunity to study the impacts of modern lifestyles on human biological rhythms and metabolism. Conversely, there are some limitations to be considered when interpreting our findings: First, the limited generalizability of the results considers the study sample size. Furthermore, its cross-sectional nature precludes inferring causality and direction of the associations. Second, due to our sample size, we may have not had enough statistical power to rule out confounders and detect the effects of some variables on the multivariate models; the wide confidence intervals for effect sizes suggest that further information is needed for greater precision. Third, as a methodological limitation, BMI is a populational measure that does not cover individual characteristics of body composition, including body fat and lean mass percentage, although it is the standard clinical measure of obesity. Future work should consider the use of other estimates of adiposity, such as waist circumference, body fat, and direct measurements of metabolic parameters that were not assessed in this study due to logistic difficulties that prevented collection, storage, and transportation of biological samples. Fourth, when we were unable to measure height and weight or there was any discomfort from participants, BMI measures were self-reported. Thus, we chose to categorize this variable into three groups for it to be more reliable. Fifth, it was not possible to assess food intake, a factor that might also be associated with obesity. Finally, we collected actimetry data in different seasons (80 participants between March and September and 41 participants between September and March). However, we included season of data (March 20 to September 22 or September 22 to March 20) collection as a covariate in the final actigraphy or MCTQ multivariate models and neither was it associated with being overweight or obese, nor did it change the results.

In summary, results from this study show an association between more fragmented rhythms of activity and exposure to light and higher BMI. Also, higher levels of urbanization and lower amplitude of light exposure were associated with increased risk of being overweight or obese. The present findings support the hypothesis that disturbed circadian rhythms and irregular light exposure might be associated with increased risk for obesity, which warrants further investigation, considering the strong potential of preventive measures.

## Data Availability Statement

The datasets presented in this article are not readily available because the datasets presented in this article are not readily available because the data in this study cannot at this stage be publicly provided. Participants were not asked for consent to make individual data available. The data contain information that could compromise the privacy of research participants. Requests to access the datasets should be directed to LKP, luisa.pilz@ufrgs.br.

## Ethics Statement

The studies involving human participants were reviewed and approved by Ethics Committee of the Hospital de Clínicas de Porto Alegre. The patients/participants provided their written informed consent to participate in this study.

## Author Contributions

RL, TR, LKP, and MPH contributed to conception and design of the study. DBC, NBX, LKP, and RL participated in data collection and organized the database. DBC, NBX, and LKP performed the statistical analyses and interpretation of data. DBC wrote the first draft of the manuscript. NBX and LKP wrote sections of the manuscript. All authors contributed to manuscript revision, read, and approved the manuscript before submission.

## Author Disclaimer

The authors alone are responsible for the content and writing of the paper.

## Conflict of Interest

TR is the founder and CSO at Chronosulting UG. None of his consulting activities in this context had any relationship with the current study. The remaining authors declare that the research was conducted in the absence of any commercial or financial relationships that could be construed as a potential conflict of interest.

## Publisher’s Note

All claims expressed in this article are solely those of the authors and do not necessarily represent those of their affiliated organizations, or those of the publisher, the editors and the reviewers. Any product that may be evaluated in this article, or claim that may be made by its manufacturer, is not guaranteed or endorsed by the publisher.
