# Supplementary material for: Relationship Between Circadian Strain, Light Exposure, and Body Mass Index in Rural and Urban Quilombola Communities
Source: Front Physiol. 2022 Jan 26;12:773969. doi: 10.3389/fphys.2021.773969 (PMC8826472; doi:10.3389/fphys.2021.773969)
Supplement: Supplementary file 1 [file Data_Sheet_1.docx]

Supplementary Material

# Supplementary Tables

| **TABLE S1:** Sample characteristics of each community | | |  |  |
| --- | --- | --- | --- | --- |
|  | **MCTQ**  **(N = 244)** | **Actimetry**  **(N = 121)** | | |
|  |  |  | | |
| **No electricity (N = 29)** |  |  | | |
| Bombas n (%) | 29 (11.9) | 28 (23.1) | | |
| **<5 yrs (N=19)** |  |  | | |
| Areia Branca n (%) | 12 (4.9) | 11 (9.0) | | |
| São João n (%) | 2 (0.8) | 0 | | |
| **>15 yrs (N = 91)** |  |  | | |
| Córrego do Franco n (%) | 13 (5.3) | 6 (4.9) | | |
| Mamãs n (%) | 24 (9.8) | 13 (10.7) | | |
| Serra do Apon n (%) | 20 (8.2) | 1 (0.8) | | |
| São Roque n (%) | 19 (7.8) | 10 (8.2) | | |
| Faxinal n (%) | 6 (2.5) | 4 (3.3) | | |
| **≥30 yrs (N = 110)** |  |  | | |
| Cantão das Lombas n (%) | 17 (7.0) | 6 (4.9) | | |
| Morro do Fortunato n (%) | 44 (18.0) | 12 (9.9) | | |
| Peixoto dos Botinhas n (%) | 37 (15.2) | 17 (14) | | |
| **Urban (N = 27)** |  |  | | |
| Areal da Baronesa n (%) | 14 (5.7) | 8 (6.6) | | |
| Quilombo Fidélix n (%) | 6 (2.5) | 4 (3.3) | | |
| Quilombo Machado n (%) | 1 (0.4) | 1 (0.4) | | |

| **TABLE S2:** Bivariate analysis of MCTQ variables, activity, light and temperature Cosinor parameters and BMI categories | | | | | | | |
| --- | --- | --- | --- | --- | --- | --- | --- |
|  | **Normal Weight**  N = 92 MCTQ  N = 49 actimetry | **Overweight**  N = 79 MCTQ  N = 34 actimetry | **Obesity**  N = 73 MCTQ  N = 38 actimetry | **p-value**  (Normal x Overweight) | **p-value**  (Normal x Obese**)** | **p-value**  (Overweight x Obese) |  |
| **MCTQ variables:** median [Q_1_ – Q_3_] |  |  |  |  |  |  |  |
| MSW (hh:mm) | 2:32 [1:49 - 3:15] | 2:49 [2:00 - 3:25] | 2:34 [1:47 - 3:19] | n.s. | n.s. | n.s. |  |
| MSF (hh:mm) | 2:56 [2:18 - 4:00] | 3:10 [2:02 - 4:15] | 2:52 [2:15 - 4:00] | n.s. | n.s. | n.s. |  |
| SD on workdays (h) | 7.96 [6.92 - 8.85] | 7.83 [6.54 - 8.83] | 7.5 [6.00 - 8.67] | n.s. | n.s. | n.s. |  |
| SD on free days (h) | 8.41 [7.25 - 9.33] | 8.33 [7.38 - 9.5] | 8 [6.83 - 9] | n.s. | n.s. | n.s. |  |
| Time spent outdoors on workdays (h) | 6 [3.25 - 8] | 6 [3 - 9] | 6 [2.75 - 9.12] | n.s. | n.s. | n.s. |  |
| Time spent outdoors on work-free days (h) | 5.0 [2.0 - 8.0] | 5.0 [2.0 - 8.0] | 5.0 [2.0 - 8.0] | n.s. | n.s. | n.s. |  |
| Social jetlag (SJL; h) | 0.25 [0 - 1.06] | 0.40 [0 - 1] | 0.00 [0 - 0.75] | n.s. | n.s. | n.s. |  |
| **Activity** |  |  |  |  |  |  |  |
| MESOR* | 283 [190 - 369] | 217 [165 - 245] | 244 [188 - 328] | **0.02** | 0.76 | 0.23 |  |
| Acrophase | 13.4 [12.6 - 14.2] | 13.5 [12.9 - 14.6] | 13.7 [13.2-14.7] | n.s. | n.s. | n.s. |  |
| **Light** |  |  |  |  |  |  |  |
| MESOR* | 670 [487-1070] | 546 [279 - 846] | 349 [265 - 693] | 0.13 | **0.002** | 0.48 |  |
| Acrophase | 12.7 [12.1 - 13.3] | 12.6 [12.3 - 13.4] | 12.7 [12.0 - 13.3] | n.s. | n.s. | n.s. |  |
| *p < .05 in Kruskal Wallis/ p-values of Dunn test (with Šidák correction). | | | | | | | |

| **TABLE S3:** Kruskal-Wallis effect sizes (epsilon- and eta-squared) | | | |
| --- | --- | --- | --- |
|  | **ε² [bootstrapped 95% CI]** | **η² [bootstrapped 95% CI]** | **Interpretation*** |
| **Activity** |  |  |  |
| IV | 0.112 [0.029 – 0.246] | 0.097 [0.01 – 0.23] | moderate |
| IS | 0.022 [0.002 – 0.118] | 0.006 [-0.01 – 0.1] | small |
| M10 | 0.077 [0.017 – 0.200] | 0.061 [0.00 – 0.19] | moderate |
| L5 | 0.021 [0.001 – 0.111] | 0.004 [-0.02 – 0.1] | small |
| RA | 0.073 [0.015 – 0.190] | 0.057 [0.00 – 0.18] | small |
|  |  |  |  |
| **Light** |  |  |  |
| IV | 0.135 [0.043 – 0.278] | 0.120 [0.03 – 0.27] | moderate |
| IS | 0.046 [0.006 – 0.160] | 0.029 [-0.01 – 0.15] | small |
| M10 | 0.103 [0.029 – 0.241] | 0.087 [0.01 – 0.23] | moderate |
| L5 | 0.077 [0.015 – 0.210] | 0.062 [0.00 – 0.20] | moderate |
| RA | 0.129 [0.042 – 0.281] | 0.115 [0.03 – 0.27] | moderate |
| *Based on rule of thumb for η²: small 0.01; medium 0.06; large 0.14 (Cohen, 1988). | | | |
|  |  |  |  |

| TABLE S4: Poisson regression: activity variables derived from actimetry associated to overweight/obesity (N= 121) | | | |
| --- | --- | --- | --- |
|  | PR | 95% CI | p-value |
| Intercept | 0.129 | 0.042 - 0.402 | 0.000 |
| Age | 1.007 | 1.000 - 1.014 | 0.067 |
| Sex (F) | 1.818 | 1.211 - 2.728 | **0.004** |
| IV (Activity) | 3.846 | 1.218 - 12.147 | **0.022** |
| M10 (Activity) | 1.000 | 0.999 - 1.001 | 0.717 |
| TABLE S5: Poisson regression: light variables derived from actimetry associated to overweight/obesity (N= 121) | | | |
|  | PR | 95% CI | p-value |
| Intercept | 0.190 | 0.109 - 0.331 | 0.000 |
| Age | 1.006 | 0.998 - 1.013 | 0.143 |
| Sex (F) | 1.781 | 1.186 - 2.674 | **0.005** |
| IV (Light) | 1.720 | 1.006 – 2.940 | **0.047** |
| L5 (Light) | 1.260 | 0.950 - 1.673 | 0.109 |

| TABLE S6: Poisson regression: variables derived from actimetry (ActTrust) associated to overweight/obesity (N= 121) | | | | |
| --- | --- | --- | --- | --- |
|  | PR | 95% CI | p-value |  |
| Intercept | 0.147 | 0.0649- 0.313 | 0.000 |  |
| Age | 1.004 | 0.996 - 1.011 | 0.349 |  |
| Sex (F) | 1.684 | 1.1397- 2.495 | **0.009** |  |
| Actimeter (ActTrust) | 0.887 | 0.649 - 1.212 | 0.451 |  |
| IV (Light) | 1.494 | 0.870 – 2.565 | 0.145 |  |
| IV (Activity) | 3.132 | 1.444 – 6.797 | **0.004** |  |

| TABLE S7: Demographic characteristics for each BMI categorie | | |
| --- | --- | --- |
| **Body Mass Index (BMI) status** | % Females |  |
| Normal weight | 41.3% |  |
| Overweight | 52.5% |  |
| Obese | 89.3% |  |

## Supplementary Figures

**Figure S1.** Median activity and light profiles of each group. The medians (bold line) and standard error (shadow) are presented for the normal weight group (orange), overweight group (light blue) and obese group (dark blue).

**
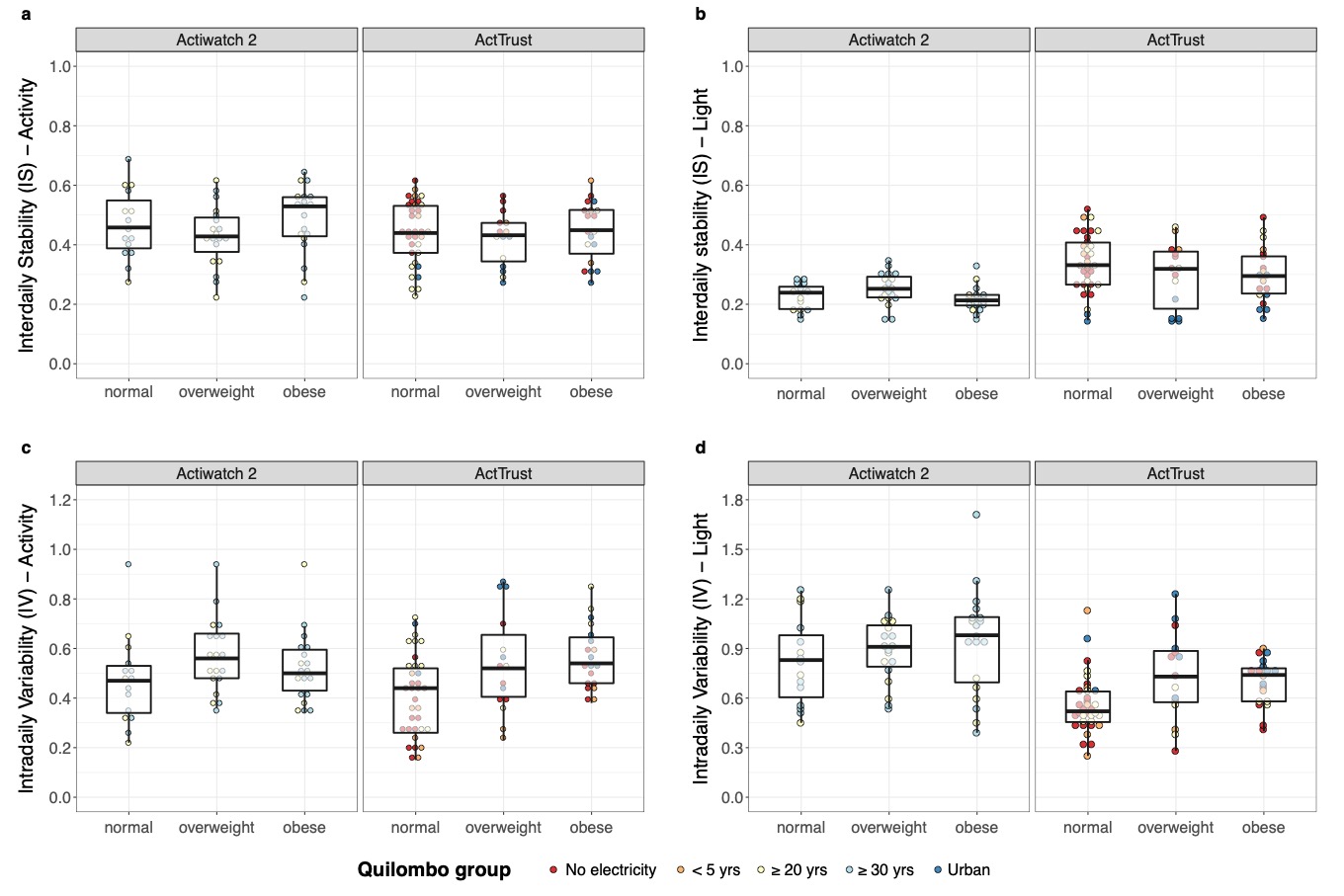
**

**Figure S2.** Activity IS (**a**), activity IV (**c**), light IS (**b**) and light IV (**d**) in normal, overweight group and obese group by actimeter brand. IS: Interdaily Stability; IV: Intradaily Variability.

**
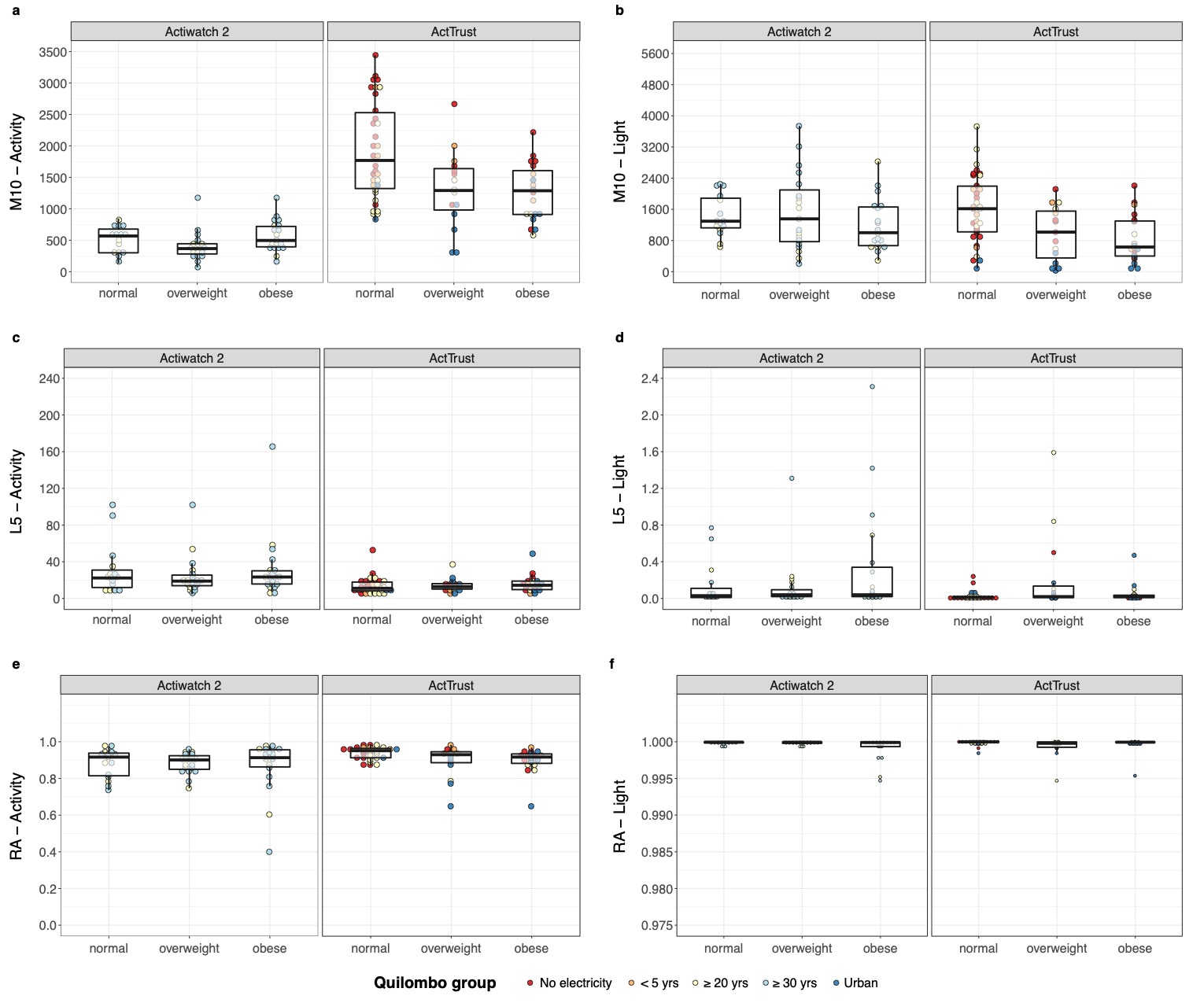
**

**Figure S3.** Activity M10 (**a**), activity L5 (**c**), activity RA (**e**), light M10 (**b**), light L5 (**d**) and light RA (**f**) in normal, overweight, and obese groups by actimeter brand. M10: mean activity/light exposure of the 10 consecutive hours with the highest values of a daily profile; L5: average activity/light exposure of the 5 consecutive hours with the lowest values of a daily profile; RA: relative amplitude. * ActTrust activity M10 and L5 were multiplied by 0.25 for scaling the plots.

**Figure S4.** Median activity and light profiles of each group in subjects wearing Actiwatch 2 (a, b) and ActTrust (c, d). The medians (bold line) and standard error (shadow) are presented for the normal weight group (orange), overweight group (light blue) and obese group (dark blue).

**Figure S5.** Examples of series with low intra-daily variability (IV).

**Figure S6.** Examples of series with high intra-daily variability (IV).

**
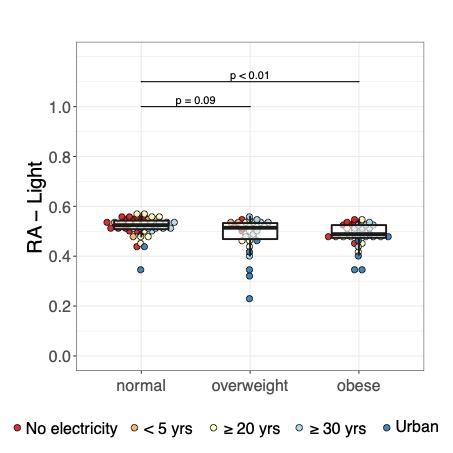
**

**Figure S7.** RA of light computed as (log10(M10+10) - log10(L5+10))/ (log10(M10+10) + log10(L5+10)). Kruskal-Wallis. P-values according to Dunn-test with Sidak correction.
